# PCSK9: A Key Target for the Treatment of Cardiovascular Disease (CVD)

**DOI:** 10.34172/apb.2020.062

**Published:** 2020-08-09

**Authors:** Saeideh Sobati, Amir Shakouri, Mahdi Edalati, Daryoush Mohammadnejad, Reza Parvan, Javad Masoumi, Jalal Abdolalizadeh

**Affiliations:** ^1^Drug Applied Research Center, Tabriz University of Medical Sciences, Tabriz, Iran.; ^2^Department of Biochemistry, Higher Education Institute of Rab-Rashid, Tabriz, Iran.; ^3^Hematology and Oncology Research Center, Tabriz University of Medical Sciences, Tabriz, Iran.; ^4^Paramedical Faculty, Tabriz University of Medical Sciences, Tabriz, Iran.; ^5^Department of Biosciences, University of Milan, Via celoria 26, 20133, Milan, Italy.; ^6^Immunology Department, Rafsanjan University of Medical Sciences, Rafsanjan, Iran.; ^7^Immunology Research Center, Tabriz University of Medical Sciences, Tabriz, Iran.

**Keywords:** Atherosclerosis, Cholesterol, Coronary heart disease, LDL, Monoclonal antibody, PCSK9

## Abstract

Proprotein convertase subtilisin/kexin type 9 (PCSK9), as a vital modulator of low-density lipoprotein cholesterol (LDL-C) , is raised in hepatocytes and released into plasma where it binds to LDL receptors (LDLR), leading to their cleavage. PCSK9 adheres to the epidermal growth factor-like repeat A (EGF-A) domain of the LDLR which is confirmed by crystallography. LDLR expression is adjusted at the transcriptional level through sterol regulatory element binding protein 2 (SREBP-2) and at the post translational stages, specifically through PCSK9, and the inducible degrader of the LDLR PCSK9 inhibition is an appealing new method for reducing the concentration of LDL-C. In this review the role of PCSK9 in lipid homeostasis was elucidated, the effect of PCSK9 on atherosclerosis was highlighted, and contemporary therapeutic techniques that focused on PCSK9 were summarized. Several restoration methods to inhibit PCSK9 have been proposed which concentrate on both extracellular and intracellular PCSK9, and they include blockage of PCSK9 production by using gene silencing agents and blockage of it’s binding to LDLR through antibodies and inhibition of PCSK9 autocatalytic processes by tiny molecule inhibitors.

## Introduction

High level of low-density lipoprotein cholesterol (LDL-C) has been continually related to risk of cardiovascular disease, especially coronary heart disease (CHD). The LDL receptors (LDLRs) on hepatic cells collude be eliminate LDL particles from peripheral blood. LDL binds to LDLR and the complicated form, LDL/LDLR, is coopted into clathrin covered vesicles through of endocytosis. After separation of LDL from the receptor, LDLR is recycled for re-use. Simultaneously, LDL is broken down. As a whole, this is a continuous process towards the plasma membrane.^1-4^

Proprotein convertase subtilisin/kexin type 9 (PCSK9) encoded via the human PCSK9 gene on chromosome 1.^5^ It is universally expressed in a lot of tissues and cells.^6^ PCSK9 binds to the receptor of LDL-C within the liver, LDLR eliminates LDL-C from the blood. While PCSK9 binds to LDLR, the receptor is broken down and might not cast-off LDL-C from the blood. If PCSK9 is blocked, extra LDLRs could be existent on the surface of the liver and will do away with greater LDL-C from the blood.^7^ Consequently, obstructing PCSK9 can decrease cholesterol levels in plasma.^8-10^ The clinical significance of PCSK9 is for acting in cholesterol homeostasis. PCSK9 is blocked by drugs, consequently decreasing LDL-C. The U.S. Food and Drug Administration (FDA) have been approved the primary PCSK9 inhibitors, evolocumab and alirocumab, for decreasing LDL-C in 2015.

The producers did not submit records to reveal that the medicine certainly advanced consequences of cardiovascular sickness, but they assumed that lowering LDLC could deal with cardiovascular disorder.^11,12^ The production cost of those new medicines as in 2015 affected their prices.^13,14^

## History

A biochemistry scientist in the Institute of Montreal in Canada, determined a singular human proprotein convertase gene located on chromosome 1 in 2003 (Nabil Seidah). Also, Catherine Boileau in the Necker-Enfants Malades clinic in Paris investigated groups with familial hypercholesterolaemia at the same time. They recognized a mutation on chromosome 1 in a number of those families; however, they were not able to discover the applicable gene. The labs were given collectively and one year later their work was posted, linking mutations within the gene, now diagnosed as PCSK9, under the given circumstance.^15^

They demonstrated that the mutations would probably lead to gene overexpression. Also, investigators at Rockefeller University^16^ and University of Texas Southwestern had detected equal protein in mice in the same year. They had demonstrated the new pathway for control of LDL-C related to PCSK9 gene expression.

Temporarily, Abifadel et al at UT-Southwestern analyzed humans with very excessive and low LDL-C and accumulated DNA samples. The approximate function of PCSK9 and its region within the genome, the applicable location of chromosome1 was sequenced in humans with very low cholesterol and nonsense mutations were observed inside the gene, hence this biological target was validated for drug treatment.^17^ Finally, the FDA authorized the PCSK9 as a novel therapeutic target in July 2015.

## Structure of PCSK9

**Figure 1:**
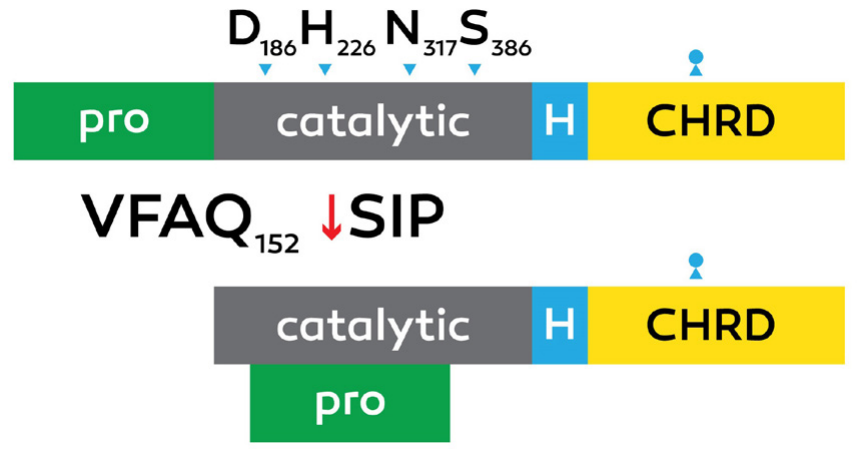
**Schematic design of proprotein convertase subtilisin kexin 9 (PCSK9) processing.** The autocatalytic zymogen processing of pro PCSK9 (75 kDa) at Val-Phe-Ala-Gln152↓Ser-Ile-pro (VFAQ152↓SIP) into the [pro segment (15 k Da) =PCSK9 (62 k Da)] complex is emphasized, collectively with the positions of the active site Asp186, His226, and Ser386 and the oxyanion hole Asn317. The C-terminal hinge area (H) and cys-his–rich domain (CHRD) are revealed.

The PCSK9 is a 72 kDa protein with 692 amino acids and composed of a prodomain with 31–152 residues, signal peptide with 1–30 residues, a C-terminal domain with 153–451 residues and a catalytic domain with 153–451 residues (Figure 1).

PCSK9 includes 9 members with the modern member of the proprotein convertase circle.^15,18^ Prepro PCSK9 undergoes signal peptidase cleavage and this cleavage is needed for the maturation and secretion of PCSK9.^1,19^ At some point in rodent improvement, PCSK9 was shown to be transiently expressed in mind centers, including the telencephalon, olfactory bulb, and cerebellum.^20,21^

Current *in situ* hybridization confirmed that PCSK9 mRNA is also ample within the embryonic umbilical artery wall, such as embryonic membranes and presumptive smooth muscle cells. In the adult, PCSK9 remains noticeably expressed in hepatocytes and much less so within the small kidney and intestine.^20,21^

## Gene and mutations

The human PCSK9 gene is found on chromosome 1 on the band1q32.3; it is made up of 13 exons.^22^ This gene generates isoforms through alternatives.^23^ Studies concerning splicing mutagenesis have shown that the sequence needed for autocatalytic cleavage is degenerate, which requires additional complex labors to become aware of the ordinary substrate(s) of PCSK9.^24,25^

Some of the “loss of function” PCSK9 mutants that will occur undoubtedly are associated with the shape of PCSK9; it is likely that these mutants influence some of PCSK9 features. The shorter versions of PCSK9 is made by three mutations that disrupting right folding and secretion.^26,27^

Several extra PCSK9 variants are related to decreased plasma LDL levels and decreased cardiovascular hazard.^28^ Transporters of the D374Y-PCSK9 have been determined to possess a five to thirtyfold higher affinity for binding to LDLR in comparison with the wild types and led to the onset of untimely CHD in advance for 10 years.^29^

In a recent study by Cohen et al, it was reported that plasma LDL-C is reduced by roughly 1.0 mmol/L by PCSK9 variations, Y142X and C679X; it was also revealed that 88% reduction in CHD prevalence is correlated with PCSK9 variations, Y142X and C679X. Some other variation, R46 L, particularly dealt with in Caucasian subjects, is correlated with an LDL-C decrease of 0.5 mmol/L along with a 47% reduction of CHD risk.^30^

## Co-regulation of pcsk9 and LDLR expression by SREBP

It is believed that sterol regulatory element-binding protein-2 (SREBP-2), membrane-bonded transcription factor regulating the cholesterol homeostasis in cells^31^ is responsible for the simultaneous expression of PCSK9 at the transcriptional level. The main SREBP isoforms expressed in mouse liver have partly overlapping but distinct gene objectives: genes associated with fatty acid synthesis are controlled by SREBP-1c. However, genes associated with synthesis of cholesterol and uptake, including Pcsk9 and LDLR are controlled by SREBP-2. The proteolytic processing of SREBPs resulted in active nuclear forms. This phenomenon occurs in the reaction that decreases cholesterol levels in endoplasmic reticulum (ER) membranes.^32^ Each SREBP in mouse liver is impeded by fasting and they can be activated by feeding.^33^ In a feeding state, SREBP-2 activity is reduced to the baseline level; but insulin stimulates SREBP-1c at each of the transcriptional and protein processing degrees.^33,34^

SREBP-1a, SREBP-1c, and SREBP-2 are three versions of SREBPs. Like SREBP-1a and SREBP-1c, SREBP-2 is synthesized as a pathfinder that joins the triplex blend made up by SREBP cleavage-activating protein (SCAP) and the ER-retention protein insulin-inducible gene accompanied by excessive levels of sterols (largely derived from LDL-related cholesterol); this complex is kept in the ER and remains an inactive pool of SREBP-2. Nevertheless, in low levels of cellular sterol, the SCAP–SREBP-2 complex Shipped to the Golgi system and after proteolytic processing the SREBP-2 is ready to secrete in the cytosol. Then, SREBP-2 transported into the nucleus and acts as a mediating factor for transcriptional activation of genes that involved in cholesterol metabolism like 3-hydroxy-3-methylglutaryl coenzyme A (HMG-CoA) synthase. This enzyme activity induces the transformation of acetyl-CoA to cholesterol, another one is the HMG CoA reductase that is the rate-restricting enzyme that is responsible for converting of HMG-CoA to mevalonate, and the LDLR32, 35, 36. In healthy individuals, fasting reduces plasma PCSK9; but they are extended post-prandially, and possess a diurnal rhythm that replicates markers of hepatic cholesterol synthesis.^29,35^ In another study, 12 healthy subjects underwent fasting for 18 hours, but it was shown that a ketogenic diet was not associated with 35% in PCSK9 plasma ranges. The results showed that prolonged fasting decreased PCSK9 sharply (-50%).^36^ Further investigations confirmed that 48-hour fasting decreased PCSK9 levels that minimized at 36 hours (58% decrease vs. fed state).^35^

It is believed that the Mediterranean diet reduced plasma PCSK9 and LDL-C to -11.7% and -9.9%, respectively, without reducing the weight^37^ PCSK9 levels were reduced by diets accompany with oleic acid, which was definitely associated with cholesterol synthesis markers (lathosterol-to-cholesterol ratio.^38^ In contrast to a saturated fat food diet, PUFA diet decreased PCSK9 and lathosterol-to-cholesterol ratio. It is believed that PUFA contributes to the development of hepatic LDL cholesterol, which leads to decreased activity of SREBP-2.^39^

## Functions of PCSK9

### Hepatic cholesterol metabolism

**Figure 2:**
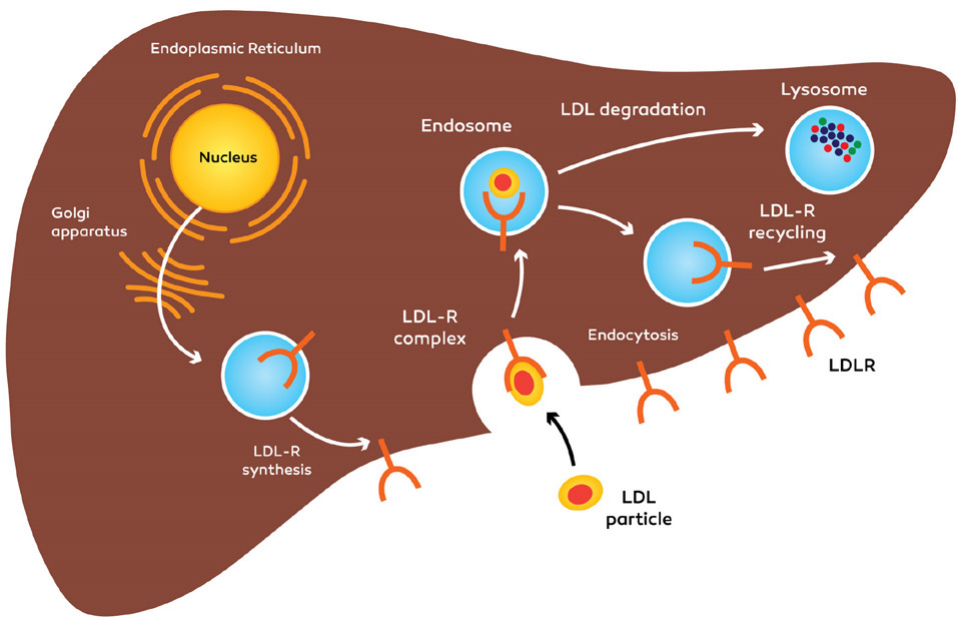
**Function of PCSK9 within the regulation of the hepatocyte LDLR activity: synthesis and recycling of LDLR.** LDLR: low-density lipoprotein cholesterol receptor, PCSK9: Proprotein convertase, subtilisin/kexin type 9.

**Figure 3:**
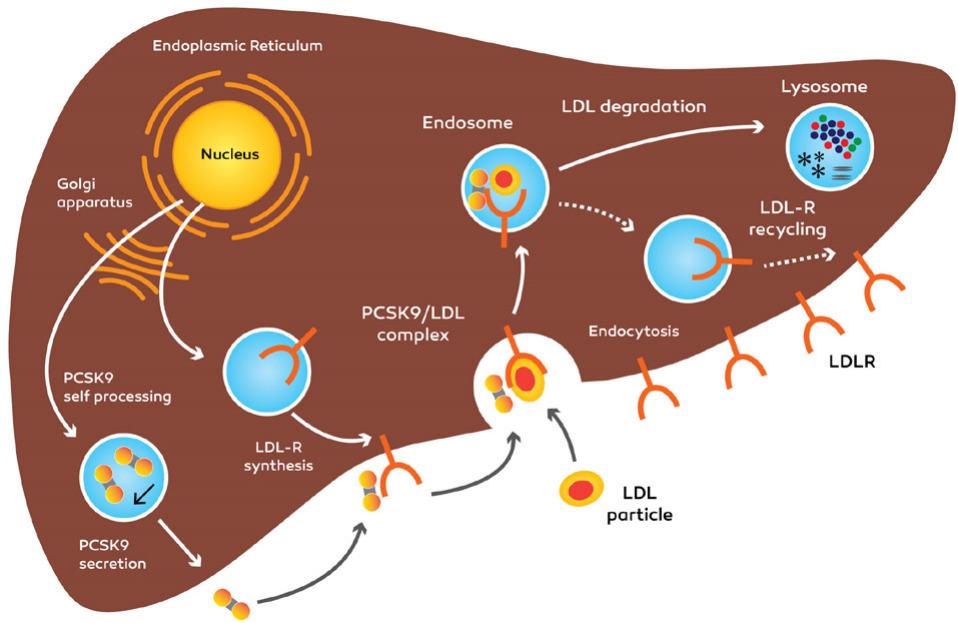
** Synthesis, secretion of PCSK9 and impact on LDLR.**PCSK9 binds to LDLR and upon internalization, directs LDLR to lysosomal degradation, reducing the quantity of LDLRs at the cellular surface. LDLR: low-density lipoprotein cholesterol receptor, PCSK9: Proprotein convertase, subtilisin/kexin type 9.

The important characteristic of PCSK9 is the degradation of LDLR through a difficult process. Through hepatic uptake, the primary part of LDL-C is eliminated of the plasma. During the endocytosis, transmembrane LDLR internalizes the LDL molecules. Internalized LDL/LDLR will be dissociated then, the LDLR returns to the cytoplasmic membrane for recycling. Every LDLR molecule is able to recycle for 150 times, it demonstrates that moderate fluctuations in LDLR accessibility via PCSK9-prompted destruction may cause significant modifications in the level of LDL-C. The PCSK9 precursor molecule experiences intramolecular autocatalytic cleavage in the N-terminal pro segment domain within the ER^40^ (Figures 2 and 3).

After PCSK9 is secreted, the cleaved pro domain keeps the connection with the catalytic domain and help mature PCSK9 molecule move out of the ER into the secretion procedure.^5,41^ PCSK9 entered to blood circulation as a phosphoprotein and has no other substrates.^29^ After PCSK9 is discharged from the cell, whether it attaches to the peripheral LDL receptor and is endocytosed collectively with the receptor, or the protein can stay in the plasma.^42,43^

The PCSK9 accomplishes plasma and entering the tissues such as the liver, kidneys, intestines, lungs, pancreas and adipose tissue, and modulate LDL-receptor recycling.^44^ One feasible reason for this apparently ineffective controlling cycle is the potential of PCSK9 as a ‘brake’ to help the gradual absorb the cholesterol while LDLRs destroyed once they have internalized LDL. PCSK9 can doubtlessly avoid extreme cellular cholesterol buildup by preventing the LDLRs recycling in the cell surface of different organs.^25^

PCSK9 attaches to the LDL receptor on the cytoplasm membrane at the first epidermal growth factor-like (EGF A) domain. The PCSK9-LDL receptor complex moves into endosomal or lysosomal space and is degraded that results in reduced LDL receptors at the surface of the cell.^45^

### PCSK9 binds the extracellular domain of the LDLR

The PCSK9 reduces the level of LDLR in hepatocytes. That observation was confirmed in mouse models for the first time then, was verified in patients affected with PCSK9 mutations through genetic tests.^26,46^ The molecular mechanism of this regression was revealed after it was shown that PCSK9 secrete by different cells and this secreted PCSK9 after attached to LDLR, undergoes internalization that speeds up its destroying.^47,48^ The internalization method is associated with the presence of ARH adaptor protein.^49^ The location of the interplay between discharged PCSK9 and the extracellular domain of the LDLR became the first epidermal growth factor-like repeat homology domain (EGF-A) inside the LDLR in the human being.

If in the EGFA domain of the LDLR a molecular change (mutation) occur, cleavage of LDLR via PCSK9 will be arrested, it is because that interacts with PCSK9 in this domain happened of the receptor. According to the current crystal structure of interaction in PCSK9/EGFA, it demonstrates that the EGF-a domain of the LDLR attaches to PCSK9 on top of the catalytic domain.^28^ Apparently, such attaching requires calcium and it is done with a 1:1 stoichiometry at a k d of 170–750 nM, at neutral pH of plasma.^29,50^ The interaction of the EGFA domain of the receptor is only with the catalytic domain of PCSK9 at the cell membrane (i.e., at neutral pH).^50^ The binding affinity for attaching the PCSK9 to LDLR is negatively regulated by the acidic stretch positioned inside the prodomain.^51^ However, the affinity between the receptor and PCSK9 is boosted (k d of 1–8 n M) than that of the neutral pH following endocytosis (i.e., at the acidic pH of endosomes).^52^ In acidic pH, salts bridges are formed by the prodomain of PCSK9 with the propeller domain of the LDLR. It was argued that charged C-terminal domain of PCSK9 attaches to the domain of the LDLR with negative charge.^53^

### Clinical significance

It demonstrated that PCSK9 substitutes are capable of decreasing or increasing upsurge of the circulating cholesterol. LDL-C is removed from the blood when it attaches to the surface of liver cells and is moved into the cells. While PCSK9 attaches to an LDLR, the receptor is wrecked along with the LDL molecule. LDLR degrades by PCSK9 through suppression of the hairpin structural modification of LDLR.^54,55^ Additional variants are associated with an uncommon autosomal dominant familial hypercholesterolemia (HCHOLA3) disease.^56,57^ Its protease activity is boosted by mutations, the amount of LDLR is reduced, and LDL cholesterol will not be absorbed into the cells.^56^ PCSK9 protein in human first undergoes transformation and settle down within the brain. However, also found in other tissue like liver, kidney, pancreas, and in small intestine.^58^ Studies have shown that PCSK9 is expressed significantly in arterial wall components such as endothelial cells, smooth muscle cells and also macrophages. Along with side effects like atherosclerosis, it would regulate homeostasis.^59,60^ Thus, it is evident that PCSK9 will involve in the pro-atherosclerotic procedure and in addition regulates lipoprotein synthesis.^30^ PCSK9 attaches to LDLR and suppress the removal of LDLC from the blood. Researchers have documents that PCSK9 inhibitors could be used for treating hypercholesterolemia conditions.^13,61-63^ Besides, loss-of-function mutations in the PCSK9 gene reduce LDL levels and safety in cardiovascular disease cases.^26,46,64^ In addition to its effect on lipoprotein synthetic procedure and its pro atherosclerotic property, PCSK9 acts as a mediator in glucose metabolism, weight complications and so on.^65-67^ The presence of PCSK9 has been observed in various types of infections (bacterial or viral) and sepsis.^68,69^ The role and features of PCSK9 in the brain are unclear; in nervous system development, it might be both pro-apoptotic and protective agent.^5^

## PCSK9 and atherosclerotic plaques

Although the majority of researches was done on the position of PCSK9 on expression of LDLR in the liver, there is increasing evidences about the expression of PCSK9 and its function in greater-hepatic tissues.^44,70^ Most recent studies show that PCSK9 as well expressed in the artery wall tissue, including macrophages, endothelium cell (EC) and smooth muscle cellular (SMC) that accompanied by localized activities that probably influence on the vascular homeostasis and formation of sclerosis in affected arthritis.^64,59^ The PCSK9 that secreted by SMC is able to degrade LDLR by macrophages; hence, it could be said that PCSK9 effects on the LDLR level inside the wall of the artery might play a role in the biology of lesion.^71^ Under the hyper lipidemic state, peripheral blood monocytes are entered into the lesion site and developed into macrophages, it leading to the development of atherosclerosis.^72^ It is believed that foam cell formation, as a main symptom of atherosclerosis, is caused by the absorption of lipoprotein by macrophages that settle down in lesion. The intake of local and unmodified LDL is facilitated by LDLR, this phenomenon only little influences on legion improvement because its feedback is regulated quickly and tightly in comparison to the huge lipid access available to scavenger receptors.^73^ Nevertheless, it was confirmed, that in C57BL/6 mouse null for macrophage LDLR that feed with specific diet causing hyperlipidemia, foam cell formation was decreased; hence, it is likely that macrophage LDLR is involved in the development of the procedure of atherosclerosis.^74^ It was shown that low stress triggers the increased PCSK9 expression in vascular EC and SMC accompanied by the production of reactive oxygen species (ROS), with phosphorylated NF-jB making a strong connection to PCSK9 expression with the mediation of LOX-1.^75^ Consequently, LDLR is detached at the surface of arterial macrophages by PCSK9 derived from SMC. This process usually triggers LDL to accumulate in the artery wall and contributes to the formation of oxidized LDL (OxLDL). Then a feed-ahead loop is set off that produces extra ROS within the cells which, in turn, causes further changes in LDL and their attachment to LOX-1.^64,75^ According to the results of the study on PCSK9 knockout mice that received PCSK9-expressing bone marrow cells, macrophage produce the PCSK9 and released into the circulation; it should be noted that less than 1% of total plasma PCSK9 is produced by macrophages.^58,59^ Moreover, secreted PCSK9 from macrophages and/or SMC are able to reduce the expression of LDLR in macrophages having a likely positional paracrine and autocrine degrade the LDLR in atheroma cells. It was reported that C57BL6 mice with macrophages expressing or missing LDLR have enhanced level of macrophage LDLR on lipid buildup in the artery wall and atherosclerotic lesion progress.^74^ Significant more small lesions have been observed in animals receiving LDLR bone marrow in comparison with those receiving normal bone marrow; hence, it could be said that the number of foam cell formation inside the artery wall is affected by macrophage LDLR, leading to atherosclerosis development ultimately. It was observed that decreased LDLR level by PCSK9 in macrophages influences on the reduced number of foam cell development inside the artery wall and causing decreased atherosclerosis manifestation.^58,76^ Hence, it could be said that PCSK9 secreted through macrophages released into the plasma and the atheroma, becomes gathered in the lesion that affects the composition of plaque regardless of the amount of serum lipid. Therefore, this could be another cardiovascular advantage of anti-PCSK9 treatment options.^60^

## Strategies for inhibition of PCSK9

Several healing methods to inhibit PCSK9 were proposed, which concentrated on both extracellular and intracellular PCSK9.^77-79^ These techniques include suppression of PCSK9 synthesis by gene silencing agents, suppression of PCSK9 attachment to LDLR by monoclonal antibodies (mAbs), and suppression of PCSK9 autocatalytic processing by small-molecule suppressors.^15,80-82^

## Antisense oligonucleotides

A common procedure to suppress the secretion of PCSK9 is to control the mRNA of PCSK9; this could be done by applying antisense oligonucleotides (ASOs); it is a quick sequence of nucleotides that attaches to the mRNA and suppress the translation procedure.^83^ Murine hepatic Pcsk9 mRNA secretion and the LDL-C level were reduced to 92% and 32% respectively by the 2nd-generation ASO for PCSK9. In addition, this kind of ASO is also capable of overexpression of Apo bec1 mRNA, a three-fold reduction within the stage of Apo B48, and a 50% reduction in the level of Apo B100.^84^ Besides, after ASOs are injected into monkeys, the serum level of PCSK9 was reduced by 85%, and LDL C level was decreased by 50%.^85^ This study demonstrated no discouraging results have been observed from the ASOs.^86^ At present, ASOs targeting PCSK9 is not being tested; a section I trial in 2011 was ended in advance because of secret reasons.

## Small interfering RNA

Other techniques for suppressing mRNA are use the single-stranded RNA or small interfering RNA (siRNA) that are probably carried out intravenously by small lipoid nanoparticles.^87^ Experiments conducted on mice models show that specific siRNA of liver concentrated on PCSK9 was able to suppress mRNA 50%-60% maximally that decreased the LDL level of plasma by 30%. In nonhuman experiments, single-dose management of five mg of the drug reduced the LDL C by 56%-70% after 72 hours and it was kept constant within some weeks.^88^ Phase I clinical trial carried out through Alnylam prescribed drugs with ALN-pcs siRNA has been done.^89^

## Monoclonal antibodies

The production process of mAbs starts in so-called hybridoma cells. The first generations of therapeutic mAbs had been produced in mouse origin cells, regularly developed the more specific human anti-mouse antibodies and ensuing reduced multiplied hazard of hypersensitive reaction.^90,91^ In the end, chimeric, humanized mAbs developed in a human consistent location, which every kind has growing extents of human sequence inside the variable location and less immunogenicity.^92,93^ Clinical administration of mAbs well-tolerated normally through intravenous and subcutaneous.^8^

The mAbs have several applications including Cancer Immunotherapy,^91,94^ Bone Marrow and Organ Transplantation,^95,96^ Protein Purification,^97-99^ Disease Diagnosis in immunoassay tests including enzyme-linked immune sorbent assay (ELISA), western blotting, immunohistochemistry and immunofluorescence tests.^100,101^ Our team has been developed the mAbs against a wide range of antigens including TNF-α,^102^ CD20,^103^ CD34,^104,105^ receptor tyrosine kinase-like orphan receptor proteins,^106^ human epidermal growth factor receptor,^107^ heat shock protein 70,^108^ vascular endothelial growth factor receptor 2,^77,78^ human IgG and IgA.^109,110^

The mAbs against PCSK9 categorized as anti-lipid drugs. Three monoclonal antibodies have been tested in terms of their efficacy and protection including Praluent (Alirocumab; REGN 727) (Evolocumab; AMBG 1451) and Bococizumab (RN 316) Alirocumab and Evolocumab, which are the main suppressor of PCSK9 determined by the FDA and EU medical researchers.^77,78,82^

On 24th of July 2015, the FDA confirmed the use of Alirocumab for diet and maximally tolerated statin therapy in adults with HeFH or patients suffering from current medical ASCVD accompanied by coronary heart attack or stroke who should undergo excessive LDLC reduction. Likewise, on the 27th of August 2015, the FDA verified the use of Evolocumab for diet and maximally tolerable statin remedy in adults with HeFH and HoFH or patients diagnosed for medical ASCVD inclusive of coronary heart attack or stroke who should undergo excessive LDLC reduction.

Bococizumab, which was developed by Pfizer, was not confirmed by the FDA. It underwent through phase two trial.^111^ Suppression of PCSK9 can be investigated based on some of the finished scientific trials such as the mAbs method as the main objective for decreasing the level of atherogenic lipoproteins. Monoclonal antibodies are capable of decreasing LDL-C, non-HDL-C and Apo B levels significantly accompanied by desirable effects on Lp(a) and sometimes TG stages.

Studies show that PCSK9 suppressors in humans influence HDL-C and Apo AI degrees minimally.

It has been shown that PCSK9 is greatly influenced by mAbs; it has also been used for statins in hypercholesterolemic patients without or with familial hypercholesterolemia, in patients intolerant to statin remedy and additionally in monotherapy.^1^ It is believed that statins are the main options for treating heightened levels of LDLC. They reduce LDLC by suppressing the enzyme that is responsible for the synthesis of cholesterol in the liver. Likewise, statins increase the expression of LDLR (as well as of PCSK9). It is believed that statin is able to decrease LDLC by 30–40% (based on the dose of statin). Nevertheless, the absolute value of LDL-C is seldom lower that <60 mg/dL. Monoclonal antibodies suppress PCSK9 and stop the destruction of LDL(R). They can decrease LDLC to 25–40 mg/dL (50%–75% reduction) and outperform statins and ezetimibe in terms of strength, and power.^111^

Recent findings show that a thoroughly completely human mAb focused on PCSK9 is probably able to decrease concentrations of atherogenic lipoproteins significantly accompanied by a considerable reduction in LDLC, Apo B, non-HDL-C and besides Lp (a) concentrations.

**Figure 4:**
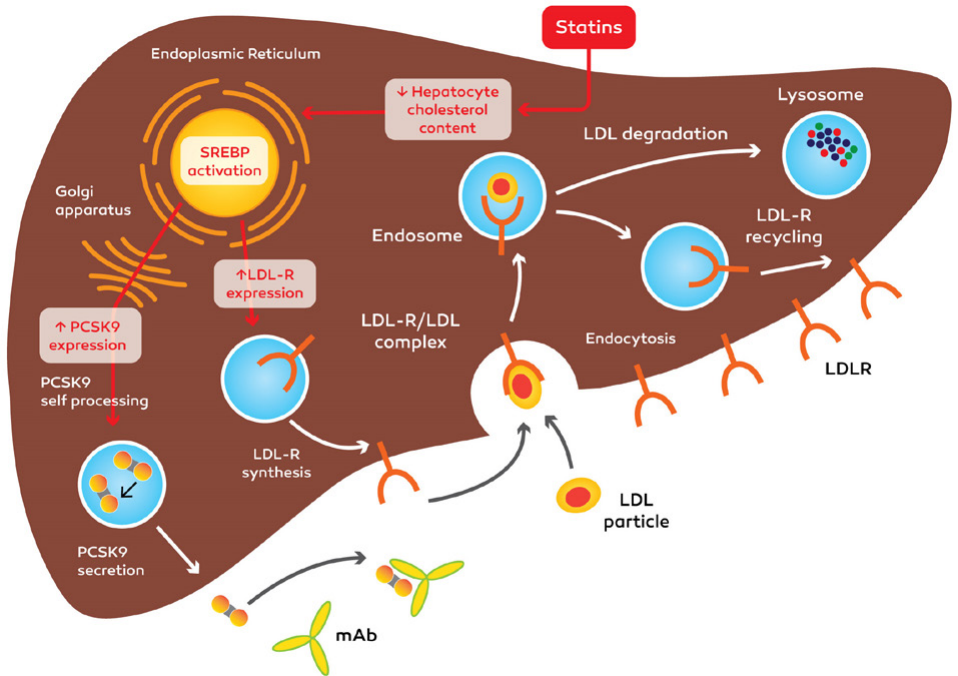
**Mechanism action of anti-PCSK9 monoclonal antibody ( mAb ) and statins.** Anti-PCSK9 mAb prevents binding of PCSK9 to the LDLR/LDL complex. PCSK9: Proprotein convertase, subtilisin/kexin type 9, LDL: low-density lipoprotein and LDLR: low-density lipoprotein cholesterol receptor.

Up to now, the efficacy of statins has been verified in hypercholesterolaemic patients with and without FH, in patients intolerant to statin treatment and in monotherapy (Figure 4). It was reported that patients with statin intolerance are influenced by PCSK9 suppressor and ezetimibe. The ability of on-goal results of PCSK9 suppressors has not been specified by modern records. It is predictable that the effects of mAbs on PCSK9 will have fewer side effects in comparison to maximum statin doses. The brief-time (12-week) protection problems have not been specified from phase II trials and the mAbs tested so far were well tolerated in comparison with the moderate injection-site reactions.^6^

## Conclusion

PCSK9 is known to be the main player in the metabolism of LDL, particularly when it boosts the degradation of LDLR in the liver. The reduced number of cardiovascular diseases in patients diagnosed with PCSK9 LOF mutations justifies the development of PCSK9 suppressors. It is believed that PCSK9 suppression is a promising objective for treatment. The main suppressers of PCSK9 are monoclonal antibodies that impede the destruction of LDL(R) accompanied by a significant decrease of LDL c in comparison statin and ezetimibe.

## Ethical Issues

This work does not contain any studies with animals or human participants conducted by any of the authors.

## Conflict of Interest

Authors declare no conflict of interest in this study.

## Acknowledgments

This study was financially supported by a grant from Tabriz University of Medical Sciences (58092).
